# Advancing cancer research: Cutting-edge insights from colorectal cancer patient-derived xenograft mouse models

**DOI:** 10.1016/j.gendis.2025.101634

**Published:** 2025-04-11

**Authors:** Yalan Lu, Xiaokang Lei, Yanfeng Xu, Yanhong Li, Ruolin Wang, Siyuan Wang, Aiwen Wu, Chuan Qin

**Affiliations:** a NHC Key Laboratory of Human Disease Comparative Medicine, Beijing Engineering Research Center for Experimental Animal Models of Human Critical Diseases, International Center for Technology and Innovation of Animal Model, Institute of Laboratory Animal Sciences, Chinese Academy of Medical Sciences (CAMS) & Comparative Medicine Center, Peking Union Medical College (PUMC), Beijing 100021, China; b Key Laboratory of Carcinogenesis and Translational Research (Ministry of Education/Beijing), Name of Department of Unit III & Ostomy Service, Gastrointestinal Cancer Center, Peking University Cancer Hospital & Institute, Beijing 100142, China; c State Key Laboratory of Holistic Integrative Management of Gastrointestinal Cancers, Beijing Key Laboratory of Carcinogenesis and Translational Research, Department of Unit III & Ostomy Service, Gastrointestinal Cancer Center, Peking University Cancer Hospital & Institute, Beijing 100142, China; d Changping National Laboratory (CPNL), Beijing 102200, China

**Keywords:** Colorectal cancer, Drug screening, Patient-derived xenograftmodel, Precision medicine, Preclinical trial, Resistance, Translational research, Tumor evolution

## Abstract

Colorectal cancer is the third most common malignant tumor globally. The current clinical therapeutic outcome is often jeopardized by the complex pathological process that is highly heterogenous among individual patients. It becomes increasingly critical for successful treatments to have diverse valid therapeutic options in clinic, which urgently demands efficient preclinical animal model to develop new drug and screen effective and safe clinical interventions. Patient-derived xenograft (PDX) mouse models, created by implanting fresh tumor tissue into immunodeficient or humanized mice, serve as a crucial resource in translational cancer research. These models closely replicate the tissue, cellular, and genetic characteristics of the original tumors, supporting their use in precision medicine, drug discovery, biomarker research, and studies of drug resistance. However, repeated transplantation can introduce genomic instability, molecular shifts, and phenotype variability. This article explores the development, advantages, limitations, and future directions of PDX models in preclinical cancer research.

## Introduction

Colorectal cancer (CRC) ranks as the third most prevalent cancer and is the second leading cause of cancer mortality worldwide.^1^^,^^2^ In 2022, China alone recorded 592,232 new CRC cases and 309,114 deaths, making up nearly half of the world's total number.^3^^,^^4^ Even recent years, a growing trend in early-onset colorectal cancer was observed in China. CRC is characterized with global CRC burden.^3^^,^^4^ Early-onset CRC cases have also risen in China.^5^ CRC development is primarily fueled by genomic instability and epigenetic modifications, which silence tumor suppressor genes and activate oncogenes. Additional CRC mechanisms are thought to involve microbial ecosystems, tumor-associated microenvironments (e.g., macrophages, cancer-associated fibroblasts), and the gut–brain axis.^6^^,^^7^ The “seed and soil” theory further illustrates how specific organs may foster metastasis.^8^ Risk factors include genetic predispositions and lifestyle factors like smoking, inflammatory bowel disease, sedentary behavior, diet, and alcohol consumption.^9^ Despite advancements in CRC prevention and treatment, disease complexity and varied patient responses continue to hinder effective management. Identifying new biomarkers and therapeutic targets is crucial for developing improved treatment options.

The rising incidence of cancer has accelerated research into anticancer drug development. Yet, despite encouraging preclinical results, only about 7% of cancer drugs entering phase I trials reach phase III approval from the U.S. Food and Drug Administration (FDA).^10^ In clinical settings, patient variability complicates treatment, even among individuals with similar molecular profiles. To optimize drug selection for CRC, researchers have classified CRC into four consensus molecular subtypes (CMS): CMS1 (MSI immune): Characterized by microsatellite instability and high immune infiltration, this subtype comprises around 14% of cases and is associated with a favorable prognosis. CMS2 (Canonical): Marked by activation of WNT and MYC pathways, representing about 37% of cases. CMS3 (Metabolic): Defined by unique metabolic patterns and KRAS mutations, comprising approximately 13% of cases. CMS4 (Mesenchymal): Noted for elevated TGFβ and VEGF expression, linked to poorer relapse-free and overall survival rates.^11^ Despite these classifications, CRC's genetic complexity and evolution often reduce drug effectiveness. First-line therapies show about a 50% response rate, which drops to around 10% for later lines.^12^^,^^13^ Resistance to initially effective drugs also arises after repeated cycles of chemotherapy, targeted therapy, or immunotherapy, underscoring the need for more refined models to explore CRC's genetic diversity and identify new biomarkers.

The patient-derived xenograft (PDX) model meets this need by transplanting fresh human tumor tissue into immunodeficient mice, thus preserving the tumor's genetic and pathological features. With an 80–90% rate of replicating clinical responses,^14^ PDX models serve as valuable tools for investigating genetic diversity. By correlating genetic traits with drug responses, PDX models help identify biomarkers and predict treatment responses. Recognizing their predictive accuracy, the U.S. National Cancer Institute (NCI) replaced the long-standing NCI-60 cell line with PDX models in 2016.^15^ This review examines the CRC PDX model, addressing its establishment (including developments in immunodeficient mice), characteristics, applications, limitations, and current research perspectives.

## Establishment of CRC PDX models

### Establishment of CRC PDX models standard procedures

**Figure 1:**
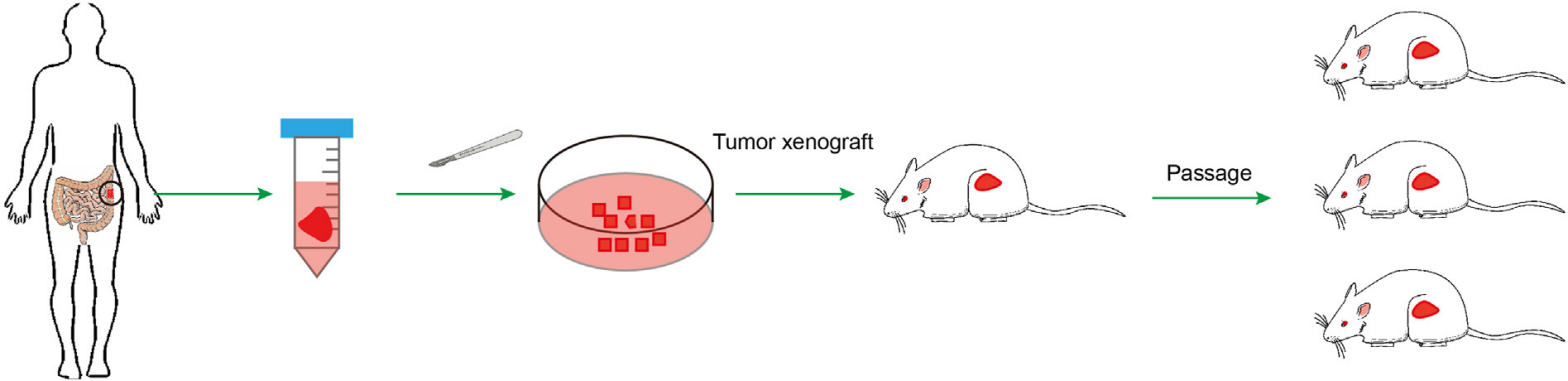
Schematic overview of CRC PDX model establishment and propagation in immunodeficient mice.

In 1969, Danish scientist Rygaard pioneered the use of human colon cancer xenografts in nude mice,^16^ initiating the development of CRC PDX models. Today's refined protocol involves collecting tumor samples during surgery, placing them in a 50 ml tube with 20 ml of DMEM supplemented with 20% FBS and antibiotics, and transporting them within 24 h under cold-chain conditions. Once in the lab, samples are washed with PBS, cut into small pieces, and promptly implanted into immunocompromised mice. Tumor growth is tracked every three days by measuring volume. When tumors reach 1 cm in diameter, they are harvested in line with ethical protocols and used to generate additional PDX models. These CRC PDX models are derived from primary, metastatic, or recurrent tumors, with recent expansions also using single-cell suspensions from effusions or circulating tumor cells. In these cases, cell pellets obtained from centrifuged seroperitoneal fluid or blood are washed and then implanted into recipient mice (Fig. 1).

### Inoculation sites

**Figure 2:**
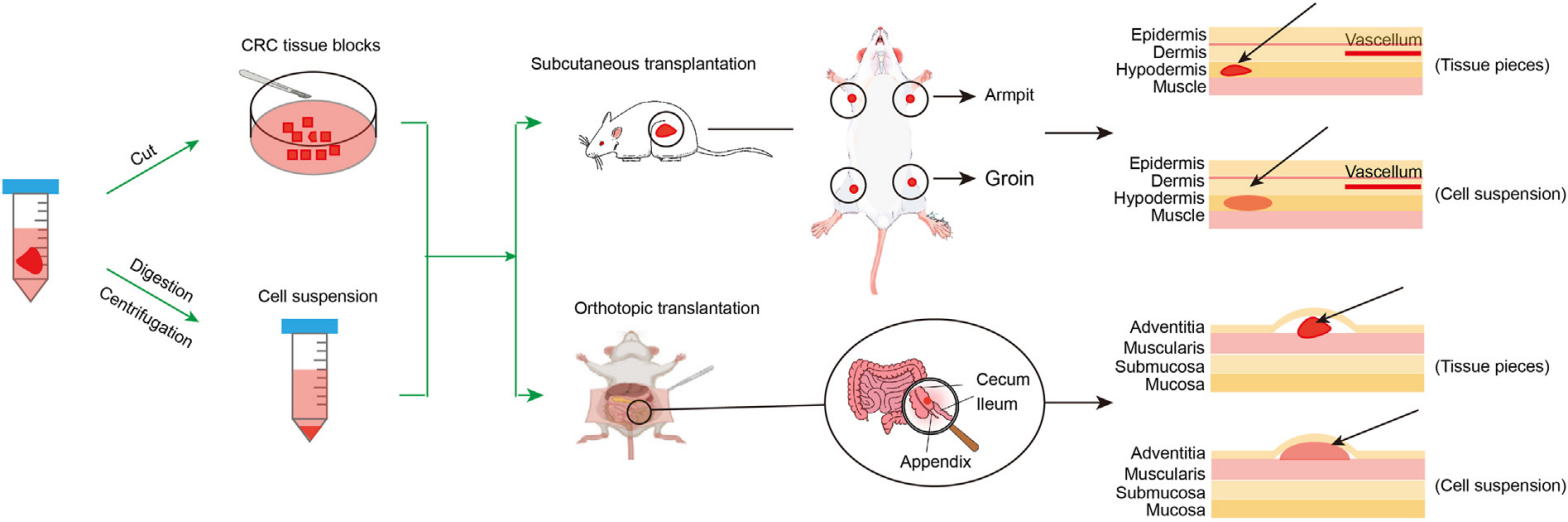
Techniques for subcutaneous and orthotopic transplantation.

Subcutaneous implantation, typically in the flank near the armpit or groin, is the preferred site, as it allows easy monitoring and supports large-scale PDX model expansion. Another approach, orthotopic injection, as used in Isabel Puig's study, introduces tumor cells into the cecum wall of NOD-SCID mice to simulate the primary tumor site and metastatic environment^17^ (Fig. 2). This method better preserves the original tumor microenvironment, achieving models with histological and genomic profiles closely matching the patient's tumor and retaining native stroma, which improves model success rates. To address the high mortality of CRC liver metastasis, researchers like Bruno Roque-Lima and Maria Laura De Angelis have developed liver orthotopic PDX models by implanting metastatic CRC tissue directly into the liver parenchyma of nude mice. These liver models faithfully reproduce tumor architecture and key molecular markers (MLH1, MSH2, MSH1, and PMS2^18^), supporting patient-specific metastasis studies and aiding clinical decisions.^19^ However, liver implantation is more complex and has a lower success rate than subcutaneous implantation due to its surgical challenges^20^ (Table 1).

**Table 1:** Comparison of subcutaneous versus orthotopic transplantation techniques.

Category	Subcutaneous transplantation	Orthotopic transplantation
Operation procedures	Simple surgery	Relative complex surgery
Operation time	Short	Long
Measure	Easy	Need animal instrument, such as nuclear magnetic resonance
Tumor formation rate	High	Higher success rate
Similarity	Similar with primary tissues	More similar histological and genomic features with primary tissues

## Development of immunodeficient mouse strains for PDX models

### BALB/c nude mice

In 1962, Grist observed naturally hairless mice within non-inbred populations, later identified as mutants with thymic dysplasia and dubbed “nude mice.”^21^ Charles River Laboratory established the inbred BALB/c-nu line by crossing BALB/cABom-nu with BALB/cAnNCrj-nu mice, producing the CAnN.Cg-Foxn1nu/Crl strain. These mice lack a thymus and T lymphocytes, resulting in impaired T cell-mediated immunity. Their hairlessness aids experimental use, but intact immune elements and occasional T cell leakage reduce tumor modeling efficiency (Table 2).

**Table 2:** Comparison of PDX model generations and associated immunodeficient mouse strains.

Category	1st generation	2nd generation	3rd generation	4th generation
Name	BALB/c nude mice	NOD/SCID mice	NSG/NOJ mice	Humanized mice
Characteristics	Lack of mature T cells	Lack of mature T and B cells; impaired NK, macrophage, and DC cells	Lack of mature T, B, and NK cells; impaired macrophage, and DC cells	Humanized immune system
Tumor formation rate	Low	Middle	High	High
Applications	Pharmacological evaluation	Pharmacological evaluation, Drug resistance, biomarkers, Tumor clonal evolution	Pharmacological evaluation, Drug resistance, biomarkers, Tumor clonal evolution	Immunotherapy evaluation

### NOD/SCID mice

In 1980, Makino et al. identified non-obese diabetic (NOD) mice, which develop type 1 diabetes through autoreactive T cells and lack functional NK, macrophage, and dendritic cells.^22^ Shortly after, Bosma et al. described severe combined immunodeficient (SCID) mice, which are devoid of functional T and B cells.^23^ In 1992, Jackson Laboratory bred NOD/SCID mice, optimizing them for PDX models in blood cancer research. These second-generation PDX models^24^ integrate the T and B cell deficiencies of SCID mice with NOD mice's additional immune deficiencies, resulting in lower rejection rates and higher transplant efficiency than BALB/c nude mice (Table 2).

### NSG or NOJ mice

**Figure 3:**
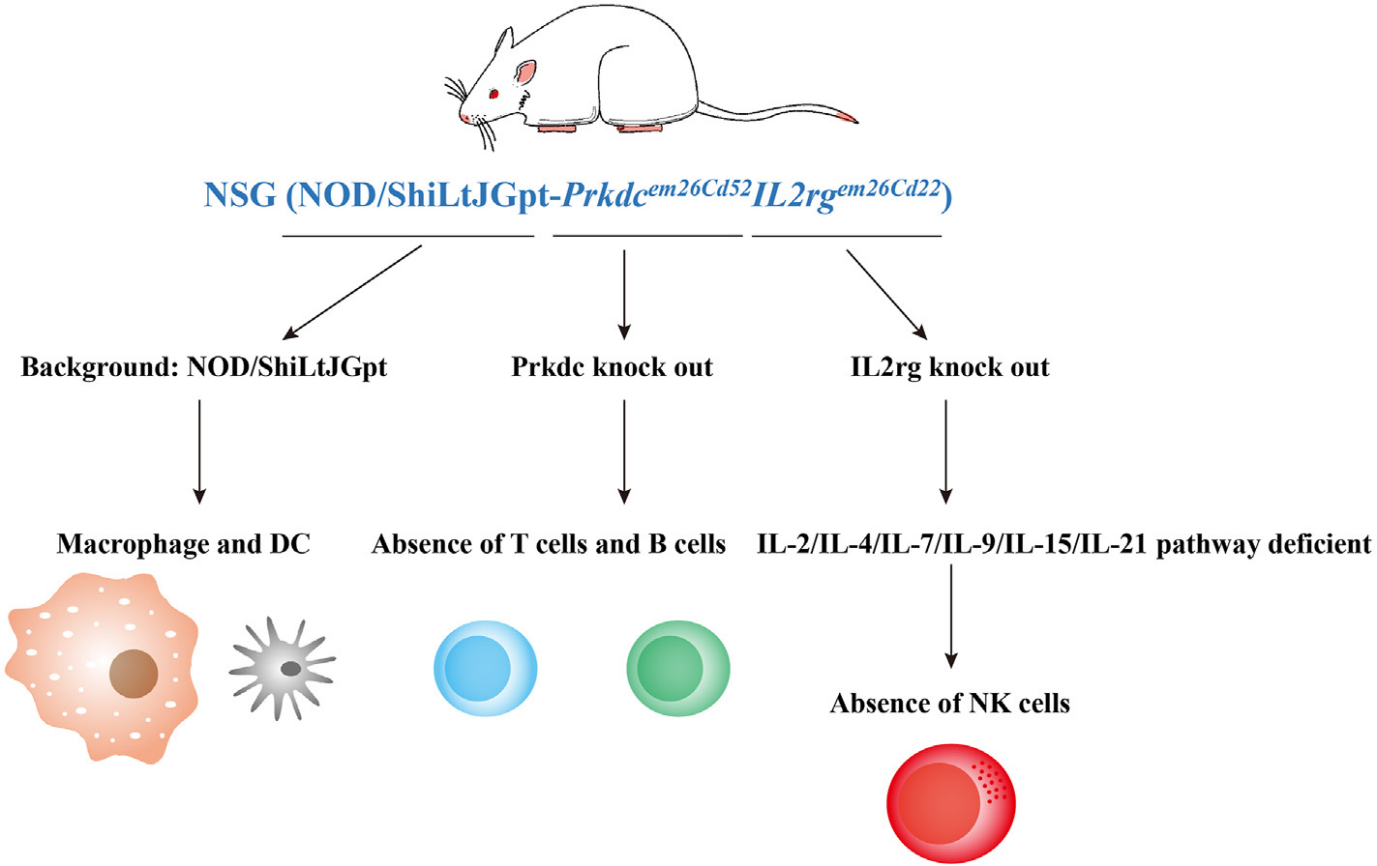
Genetic background and immune cell profile of NSG mice.

To refine NOD/SCID-based immunodeficient models, researchers bred IL2Rγ-deficient mice with NOD/SCID mice, eliminating residual NK cells. IL2Rγ, a shared receptor for several interleukins, is critical for NK cell and lymphocyte development. This cross resulted in the NOD/SCID/IL2Rγ^nul^ (NSG) strain, which lacks functional T, B, and NK cells and exhibits deficient macrophage and dendritic cell activity^24^ (Fig. 3). Similarly, NOD/SCID/Jak3^null^ mice, or NOJ mice,^24^ exhibit comparable immune deficiencies due to Janus kinase 3 (Jak3)'s role in IL2Rγ signaling. NOJ mice offer improved engraftment efficiency and are widely utilized in current research (Table 2).

### Humanized mice

Standard immunodeficient models, like NSG and NOJ, lack functional immune cells, limiting their suitability for immunotherapy studies. To address this, “humanized” mice were developed with a reconstituted human immune system.^25^ These highly immunodeficient mice are irradiated and implanted with umbilical cord blood stem cells, CD34-positive cells or bone marrow–liver–thymus tissues, allowing reconstitution of human hematopoietic lineages, including T and myeloid cells. Transplanting PBMCs into their spleens enables the development of functional human B and T lymphocytes, while NK and gamma-delta (γδ) T cells can be reconstituted in NSG mice to assess antitumor effects.^26^^,^^27^ These advanced humanized models are instrumental for cancer immunotherapy research (Table 2).

## Characteristics of CRC PDX models

**Figure 4:**
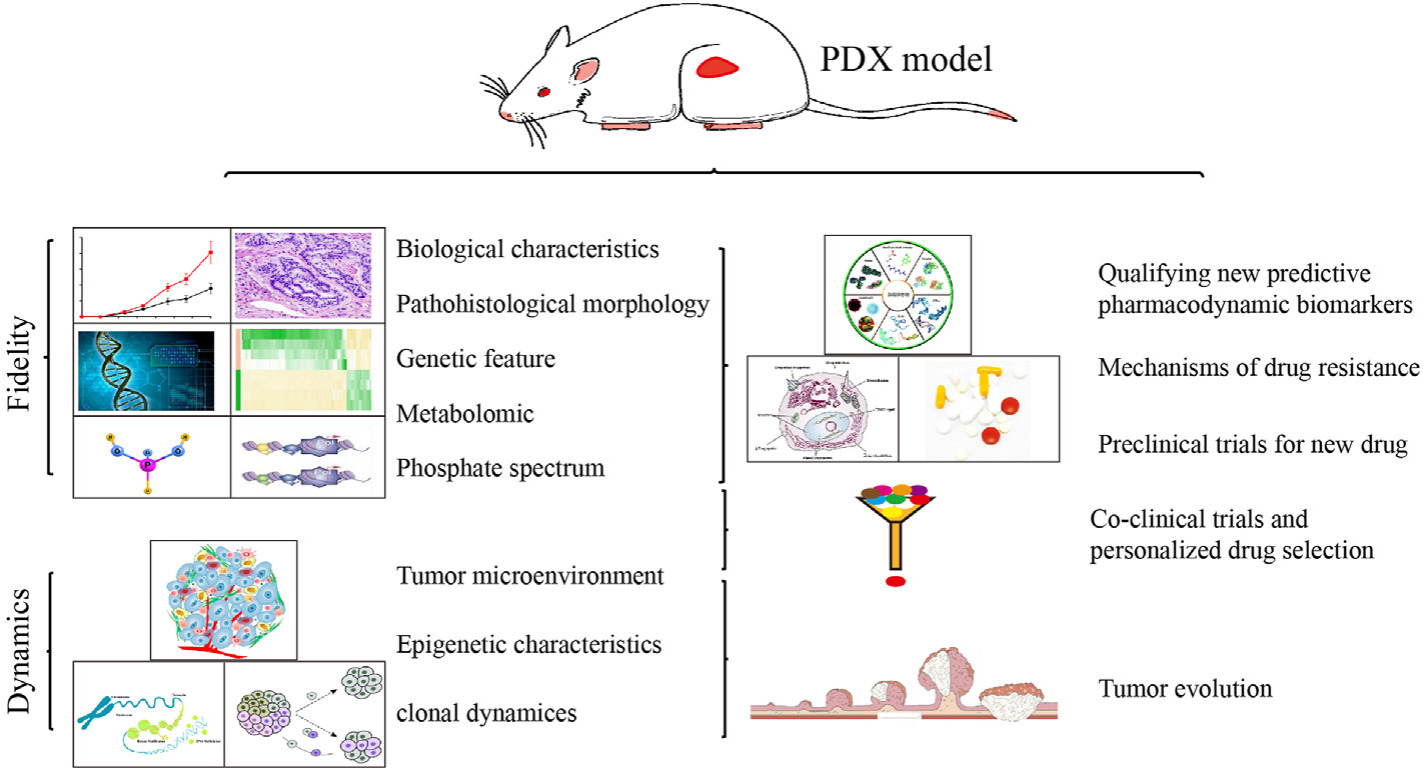
Key characteristics and applications of CRC PDX model.

CRC treatment effectiveness is often hindered by complex, variable pathology across patients. PDX models closely replicate the biological, histological, and molecular traits of primary tumors, while serial passaging preserves the tumor microenvironment (TME) and clonal diversity (Fig. 4 and Table 3).

**Table 3:** Characteristics of patient-derived xenograft models in colorectal cancer.

First Author	Year	Number	primary	metastasis	engraftment	Time for P1	Mouse strain	location
Fichtner I^1^	2004	35	33	2	43%	–	Balb/C nude mice	Subcutaneously
Dangles-Mariel V^52^	2007	26	10	16	77%	–	Balb/C nude mice	Subcutaneously
Mischek D^53^	2009	10	0	10	60%	–	SCID/beige	Subcutaneously
Linnebacher M^54^	2010	48	48	0	72%	–	NOD/SCID and Balb/C nude mice	Subcutaneously
Bertotti A^55^	2011	150	0	130	87%	–	NOD/SCID	Subcutaneously
Julien S^56^	2012	54	35	19	64%	59.4 days	Balb/C nude mice	Subcutaneously
Aytes A^57^	2012	41	41	0	89.1%	–	Balb/C nude mice	Orthotopic
Monsma DJ^58^	2012	17	17	0	47%	–	Balb/C nude mice	Subcutaneously
Chou J^59^	2013	50	34	16	58%	–	NSG	Subcutaneously
Isabel Puig^17^	2013	32	32	8	84.4% (P)	68 ± 34 days (P)	NOD/SCID	Subcutaneously
					100% (M)	46.6 ± 21.7 days (M)		
Lee W–S^60^	2014	10	10	0	100%	–	NOD/SCID and Balb/C nude mice	Subcutaneously
Cho YB^61^	2014	143	–	–	67%	90 ± 20 days	Balb/C nude mice	Subcutaneously
Bo Young Oh^28^	2015	241	191	50	62.2%	–	Balb/C nude mice	Subcutaneously
Suad M Abdirahman^30^	2020	33	32	1	66.7%	Less than 100 days	NSG	Subcutaneously
Yanmei Zhang^62^	2020	18	10	8	88.9%	50.8 ± 5.1 days	NSG	Subcutaneously
Maria Rivera^34^	2021	49	27	22	56%	49.4 ± 19.5 days	NSG	Subcutaneously
Stephanie Matschos^29^	2021	261	236	25	64.0%	Average 120 days	NSG	Subcutaneously

P: primary tumor; M: metastasis tumor.

### Biological properties

At Sungkyunkwan University, Bo Young Oh and colleagues developed a biobank of 50 PDX models from matched primary CRC and metastatic liver tumors. They found that metastatic tumors showed higher tumorigenicity than primary tumors (84.0% *vs*. 78.6%).^28^ Successful primary tumor engraftment was associated with advanced cancer stage, poorer differentiation, elevated preoperative carcinoembryonic antigen levels, vascular or lymphatic invasion, and microsatellite instability. Tumorigenicity also predicted relapse risk in stage III CRC. Similarly, Stephanie Matschos' team created a biobank of over 100 CRC PDX models,^29^ identifying notable associations between PDX characteristics and original tumor profiles. Molecular subtype influenced the time to reach harvestable size: MSI-H tumors, including sporadic and Lynch-associated cases, generally grew in less than 90 days, while other subtypes took 90–180 days. The subtype also impacted the number of mice needed, with MSI-H models requiring fewer than three mice, while CIMP-low and non-MSI types needed over 4.5 mice. Tumors from the right colon typically reached target size within 90 days, compared to 90–180 days for tumors in other locations. Furthermore, freshly passaged PDX tissues grew faster than frozen-passaged ones.^29^ These findings underscore the CRC PDX model's ability to reliably reflect the biological characteristics of patient tumors.

### Histopathological features

CRC PDX tumors largely maintained the histological features of their original patient adenocarcinomas across stages I – IV.^30^ In a study of 108 cases, 95.4% (103 cases) of PDX tumors closely matched the structural characteristics of the patient tumors, with only 4.6% (5 cases) exhibiting significant differences due to lymphoma transformation.^29^ H&E staining showed that P2-generation PDX tumors retained a structure similar to primary tumors, and immunohistochemistry indicated strong AFP, CEA, and E-cadherin expression in both PDX and patient samples.^31^ PDX tumors from six cases with distinct molecular subtypes displayed consistent morphology across animals, mirroring patient data on necrosis levels.^29^^,^^32^ Highly necrotic PDX tumors continued to exhibit this feature in later passages. Independent studies, such as those by Mingyue Li and Maria Rivera, found that H&E-stained PDX sections accurately reflected primary and metastatic tumor characteristics, allowing PDX models to represent diverse clinical profiles seen in patients.^33^^,^^34^ Research by John J. Tentler's group showed that CRC PDX models preserved histological and phenotypic traits of the original tumors for up to 14 passages.^32^ However, a study by Hirotaka's team noted that while PDX models with germline mutations retained the original tumor's histology and genetics, those with MLH1 promoter hypermethylation lost this modification, leading to structural differences.^35^ In summary, CRC PDX tumors, aside from lymphoma transformations, display stable pathomorphology across multiple passages, reliably reflecting the genetic and histological features of the original tumors.

### Genetic and epigenetic profiles

In 2021, Woo, Giordano, and colleagues analyzed copy number alterations (CNAs) in over 1400 samples from 509 PDX models and their corresponding patient tumors. They found that PDX-specific CNAs did not exhibit significant cancer-related gene enrichment and that CNA differences between patient and PDX tumors were similar to those observed across different regions within a single patient's tumor.^36^ Rivera's team also evaluated mutations in three PDX models, noting stable mutation profiles in key oncogenes and tumor suppressor genes (APC, KRAS, PIK3CA, TP53) across xenografting and passaging.^34^ Meanwhile, Tentler's group found additional, non-original mutations in some PDX tumors, though these models still preserved key features like clonal heterogeneity, chromosomal instability, and histological characteristics, even after numerous passages in CRC tumors with chromosomal instability. Unfortunately, they did not compare the mutation profiles between early and late passages in detail.^32^ In 2019, Pramudityanti's study on genetic variations across the four CMS of CRC highlighted a subtype-specific bias in PDX representation, with CMS2 particularly underrepresented.^10^ They proposed that Ki67, a proliferation marker, could enhance patient classification, prognostic evaluation, and treatment planning for CMS2 patients.^10^

In another study, Hirotaka Suto and colleagues developed two MSI-H CRC PDX models. Although one patient tumor displayed MLH1 promoter hypermethylation, the PDX models lost both this hypermethylation and MSI-H/dMMR status.^35^ Conversely, Grasse and Lienhard found that PDX methylation patterns in lung cancer models closely matched those of primary tumors, preserving specific epigenetic profiles.^37^ Finally, Kun Xiang's team profiled transcriptomic and chromatin accessibility in 11 CRC primary tumors and their corresponding PDX, PDO, and PDOX models.^38^ Chromatin accessibility patterns were largely retained across these models, with PDX and PDOX models more similar to each other in chromatin remodeling than to PDO models, likely due to their distinct microenvironments. These chromatin characteristics influence cell growth and drug sensitivity, underscoring the significance of microenvironmental factors in clinical outcome predictions.^38^

### Metabolomic profiles

Arnaud Blomme's team established six PDX models from colorectal cancer and liver metastases. By the second generation, murine cells had replaced human stromal cells, but the metabolic profiles of both stromal and cancer cells remained consistent with patient samples for at least four generations. This suggests that human cancer cells in PDXs may prompt murine stromal cells to adopt human-like metabolic characteristics.^39^ Metabolomics analysis identified two primary metabolic groups in these models—one enriched in nucleotides and carbohydrates, the other in lipids and fatty acids—regardless of breast cancer histologic subtypes. Tumor cells in PDXs more closely matched patient tumor metabolism than in vitro cultures.^40^ In related work, Yongtao Liu's analysis of the urinary proteome in colorectal PDX models revealed tumor-derived proteins in mouse urine, although immune-response proteins, potential early tumor biomarkers, were absent.^41^ Similarly, Chen, Yang, and Guo documented metabolic shifts in malignant pleural mesothelioma PDX models, specifically in amino acid metabolism, the TCA cycle, glycolysis, and nucleotide metabolism.^42^

### Phosphorylation patterns

Robin Beekhof and team conducted phosphoproteomic and proteomic profiling on 30 CRC PDX models. Their findings revealed that cetuximab-sensitive models showed increased MAPK pathway inhibition and high tyrosine phosphorylation in cell junction proteins. In contrast, cetuximab-resistant models displayed elevated MAPK and AKT signaling, epithelial–mesenchymal transition (EMT) traits, and increased Src and ephrin kinase activity. *In vivo*, targeted inhibition of these kinases, alone or in combination, effectively suppressed tumor growth.^43^ This study underscores phosphoproteomics in PDX models as a valuable tool for identifying CRC biomarkers and therapeutic targets associated with EGFR inhibitor sensitivity and resistance. In glioblastoma (GBM), Kyung-Hee Kim's proteomic and phosphoproteomic analyses revealed that recurrent tumors shift to a neuronal-like state marked by WNT/PCP signaling and BRAF kinase activation, with similar evolutionary changes seen in PDX models. BRAF inhibition suppressed neuronal transition and migration in recurrent GBM cells, key factors in post-treatment progression. Combining temozolomide with the BRAF inhibitor vemurafenib extended survival in PDX models,^44^ with similar benefits observed in models of acute lymphoblastic leukemia, neuroblastoma, and breast cancer.^44^, ^45^, ^46^

### Tumor microenvironment and clonal dynamics

The TME in CRC PDX models, including the extracellular matrix (ECM) and stromal cells such as endothelial cells, pericytes, fibroblasts, and immune cells, shows limited stability across generations. Blomme et al. found that murine stromal cells quickly replace the human stromal components after tumor implantation, with complete replacement by the second generation.^39^ Similarly, Nguyen and Yoshida observed that although early-passage PDX models retain some patient-derived stromal elements and acidic conditions, these human components are eventually supplanted by a mouse-derived TME, while the organization of epithelial and stromal cells remains similar to the original tumors.^47^^,^^48^

CRC PDXs maintain key tumor cell characteristics but undergo clonal changes over serial passages. Blomme et al. observed a loss of the differentiation marker CK20 in CRC cells starting from the third generation, signaling phenotypic shifts. In a study of 20 CRC PDXs, Cybulska's group identified an average of 0.14 variants per gene per sample,^39^ with clonal diversity decreasing as passages progressed, reflected by an increase in mutations related to cell division, ECM organization, immune responses, and angiogenesis.^49^ Using deep sequencing on primary CRC tumors, metastases, and corresponding PDXs from 11 patients, Dang et al. revealed complex clonal evolution and selective pressures influencing metastasis and PDX clonal structure.^50^

These fidelity shifts in PDX models are driven by several factors. Malignant clones, which proliferate more readily, dominate over time, leading to reduced genetic diversity and increased aggressiveness compared to the original tumor.^51^ Moreover, the use of immunodeficient mice for PDX establishment facilitates the replacement of human ECM and stromal cells with murine TME, altering cell polarity, signaling, and migration. These changes impose selective pressures, encouraging tumor subclones to adapt to the murine environment.^47^ Additionally, factors such as genomic variability, gene expression differences, and sampling bias contribute to tumor evolution in PDX models.

## Applications of PDX models

### Co-clinical trials and personalized drug selection

PDX models replicate the structural and biological characteristics of primary tumors, providing a critical platform for assessing cancer drug efficacy. A typical approach involves establishing a PDX model, treating it with various drug combinations, and tracking tumor growth and body weight changes over time. Drugs that show optimal efficacy with low toxicity are advanced for clinical consideration. At Johns Hopkins University, Izumchenko et al. compared therapeutic responses in 92 advanced solid tumor PDX models with clinical patient outcomes across 129 treatments. They found the PDX model screens had 96% sensitivity and 70% specificity, with confidence intervals (CI) of 89% and 54%, yielding positive and negative predictive values of 85% and 91%, respectively. In CRC PDX models, 85% of therapeutic responses (17 of 20 cases) aligned with patient outcomes, confirming PDX models' reliability in mirroring clinical treatment responses. A separate study demonstrated similar predictive accuracy with 15 CRC PDX models treated with 5-fluorouracil, oxaliplatin, or irinotecan, showing high correlation with patient response rates, reinforcing PDX models’ utility in guiding therapeutic selection.^14^ At UT MD Anderson Cancer Center, Timothy P. DiPeri and team developed 19 PDX models for HER2-positive cancers (esophageal, colorectal, gastric/GEJ, and breast). Among these models, 60% of patients achieved partial response or stable disease, with Zanidatamab showing concordant efficacy in xenografts and clinical responses.^63^ In cases where resistance to Zanidatamab emerged, *MET* and *MYC* gene amplifications were observed. Combining MET inhibitors with Zanidatamab extended tumor regression.^63^ These studies illustrate the predictive strength of PDX models in aligning xenograft and patient treatment responses, thereby informing clinical treatment choices (Fig. 4).

### Validation of predictive pharmacodynamic biomarkers

CRC PDX models, which closely mimic primary tumors, offer a more effective approach for identifying predictive biomarkers than traditional cell lines. Examining pharmacodynamic biomarkers for treatment effectiveness facilitates precision medicine by enabling patient-specific therapies. Large-scale PDX studies, featuring unique molecular characteristics like gene copy numbers, mutations, gene expression, and proteomic data, are essential for evaluating tumor drug responses. Specific genes or gene combinations linked to drug sensitivity have become key biomarkers. For example, Rivera et al. at Charité–Universitätsmedizin Berlin tested 49 CRC PDX models with standard drugs and found a strong association between B-RAF, EGFR, and KRAS copy numbers and sensitivity to cetuximab and erlotinib.^34^ Similarly, Bertotti et al. showed that mutations in *KRAS*, *NRAS*, and *BRAF* predict cetuximab resistance, revealing HER2 amplification in cetuximab-resistant tumors that are wild-type for KRAS, NRAS, BRAF, and PI3K. This led to “xenopatient” trials testing cetuximab combined with pertuzumab or lapatinib in HER2-amplified, cetuximab-resistant tumors. These findings demonstrate the potential of integrating genomic data with PDX models to drive drug development, biomarker discovery, and clinical applications in CRC. Building PDX biobanks with genetic and drug–response profiles allows researchers to use computational tools to identify precise drug biomarkers, enhancing personalized cancer treatment (Fig. 4).

### Investigating drug resistance mechanisms

Therapeutic resistance, both intrinsic and acquired, remains a major barrier in effective cancer treatment.^64^ For instance, although 5-FU-based therapies are initially beneficial for many CRC patients, resistance often develops, leading to poor outcomes in advanced cases.^65^ PDX models, which accurately replicate patient drug responses, are invaluable for investigating mechanisms of resistance. In an analysis of 23 CRC PDX models, Krumbach et al. formulated a “cetuximab response score” by assessing biomarkers such as epiregulin, amphiregulin, and levels of EGFR, MET, AKT, and HER3.^66^ High levels of EGFR, epiregulin, or amphiregulin increased the score, while *KRAS*, *NRAS*, or *BRAF* mutations or elevated activated MET, HER3, or AKT reduced it.^66^ Sandra Kendzia's research linked IGF2BP2 expression to chemoresistance against drugs like 5-fluorouracil, oxaliplatin, and gefitinib, suggesting IGF2BP2 as a potential target to counteract resistance.^67^ Similarly, Jiatong Lin et al. identified a CircPDIA3/miR-449a/XBP1 pathway that inhibits pyroptosis through GSDME-C domain to induce chemoresistance of colorectal cancer.^68^ For bevacizumab resistance, elevated histone lactylation was shown to upregulate RUBCNL/Pacer transcription, enhancing autophagosome maturation via BECN1 and PI3K complex activity under hypoxic conditions. The combination of bevacizumab with inhibitors of histone lactylation and autophagy significantly suppressed tumor growth in resistant PDX models.^69^ These studies highlight the crucial role of PDX models in elucidating CRC resistance mechanisms and in testing targeted therapies to address these obstacles (Fig. 4).

### Tumor evolution studies

Tumor profiles evolve dynamically under environmental pressures, and in murine hosts, the tumor microenvironment drives selection pressures that closely model human tumor evolution.^70^ Cancer stem cells (CSCs), a subset with self-renewing and differentiating abilities, show high resistance to chemotherapy and radiation, and can be isolated from PDX tumors using specific markers.^71^^,^^72^ In a study by Ha X., PDX models from 11 metastatic colorectal cancers were analyzed for clonal evolution relative to primary tumors. In four of nine xenografts, dominant clones from the original tumors became minor, while previously minor subclones grew dominant. Only two xenografts retained all original subclones, while six lost 20%–67%,^50^ suggesting that single biopsies might miss key treatment-related mutations, such as those in KRAS or PTEN. Jong-Il's study of 72 serially transplanted PDXs from 35 colorectal cancer patients found strong correlations between mutation allele frequencies and somatic copy number alterations in PDXs and primary tumors, with Pearson coefficients of 0.60 and 0.57. PyClone analysis indicated that each tumor had two to six major subclones that persisted and expanded in the PDX models.^73^ Further study of five PDX models across multiple organs showed that primary and PDX tumors maintained consistent evolutionary patterns, as reflected in stable somatic mutations, mRNA expression, and DNA methylation.^73^ Overall, PDX models effectively retain the molecular characteristics of original tumors and capture the evolutionary paths of primary and metastatic cancers, while developing some unique molecular features distinct from patient tissue (Fig. 4).

### Preclinical drug development

Preclinical studies reveal that only about 7% of tested agents are effective in clinical trials.^74^ In 2016, recognizing that PDX models more closely replicate primary tumor genetics and histology than traditional NCI-60 cell lines, the U.S. National Cancer Institute adopted PDX models for anti-cancer drug testing.^15^ Although PDX models have been used to advance drug research for CRC, no CRC-specific therapies have yet received approval. However, PDX models have supported the approval of drugs for other cancers, including AG-221 and SH1573 for IDH2 R140Q-mutant acute myeloid leukemia (AML) and elacestrant for ER^+^ breast cancer.^75^, ^76^, ^77^, ^78^ Mutations in IDH1 and IDH2 genes are common in several cancers, including AML, where they disrupt blood cell differentiation and promote leukemogenesis.^79^ IDH2 mutations occur in about 9% of AML cases, with 75% of these cases involving the IDH2 R140Q variant, making it a key target for AML treatment. AG-221, an oral selective inhibitor from Agios Pharmaceuticals, targets the mutated IDH2 enzyme. High-throughput screening confirmed AG-221's efficacy and favorable drug properties.^80^ In survival studies with IDH2 R140Q-mutant AML PDX models, AG-221 extended survival significantly compared to untreated and Ara-C-treated groups. Additional studies demonstrated dose-dependent responses and increased CD15 expression in leukemia cells, leading to FDA approval of AG-221 for relapsed or refractory AML in adults with IDH2 mutations.^76^, ^77^, ^78^ Similarly, SH1573 was approved for clinical trials in China for the same mutation target in AML^75^ (Fig. 4).

## Limitations of CRC PDX models

While PDX models offer critical benefits for CRC research by preserving the genomic, histologic, and pharmacologic traits of original tumors, they have significant limitations.

### Limited engraftment efficiency

Although CRC PDX models achieve relatively high engraftment rates (60%–90%) compared to tumors like uveal melanoma (28%) and breast cancer (19%–21%)^81^, ^82^, ^83^ success is impacted by factors such as tumor malignancy, cell density, transport conditions, handling practices, mouse strain, and environmental variables. Additionally, low success rates in cryopreservation and reanimation can lead to loss of valuable samples.^84^ Despite improvements, a 100% engraftment rate remains unattainable, limiting PDX studies to successful specimens only.

### High time, labor, and financial costs

PDX modeling demands extensive time, labor, and cost. Establishing PDX models requires tumor implantation, *in vivo* expansion, and serial passaging, with the P2–P4 generations typically used for drug testing. Each generation takes 3–6 months to grow, plus 1–2 months for drug response evaluation, resulting in a process that spans nearly six months—making PDX models unsuitable for rapid drug screening in urgent clinical situations.^85^ Both subcutaneous and orthotopic implantations involve surgical procedures, necessitating skilled personnel. Standard host NSG mice, which are highly immunodeficient, are costly and require stringent housing conditions. Immune-response studies further elevate costs, as humanized mice and specialized SPF facilities are required. In summary, while PDX models are invaluable in cancer research, limitations in engraftment success, time demands, and costs constrain their widespread use.

### Risk of lymphoma development

CRC PDX models are prone to developing lymphocytic tumors. In a study by Suad M. Abdirahman et al., 18.2% (4/22) of CRC PDX models formed lymphocytic tumors, with an additional 18.2% exhibiting adenocarcinoma and/or lymphoma,^30^ marked by CD45+ lymphocytes and atypical large lymphoid cells. Lymphoma formation has also been reported in other cancers, including head and neck (32%), ovarian (11.1%), and gastric cancers (20%),^86^, ^87^, ^88^ as well as oral squamous cell carcinoma and glioma. This likely arises from the lack of immune surveillance in immunodeficient mice, combined with Epstein–Barr virus (EBV)-induced B-cell proliferation in the tumor tissue, leading to B-cell lymphomas.^81^

### Intrinsic clonal dynamics

PDX models inevitably undergo changes in clonal dynamics, tumor evolution, and stromal composition.^39^^,^^89^^,^^90^ While PDX models aim to replicate the histopathology, genomics, and drug response of primary tumors, they accumulate genetic alterations during passaging, often due to the expansion of minor clones and adaptation to the murine TME. Many chromosomal copy number alterations observed in primary tumors diminish, while clones with malignant and CSC features predominate. Additionally, human stromal components—such as cancer-associated fibroblasts, endothelial cells, and tumor-associated macrophages—are progressively replaced by murine stroma. This shift complicates the use of PDX models to predict drug responses, as the impact of murine-derived stroma on human cancer cells remains unclear.

## Future perspectives

This review highlights the importance of PDX models in CRC research, focusing on their utility as preclinical *in vivo* models and challenges associated with their use. Early-passage PDXs, which closely resemble original tumor tissue, are highly suited for *in vivo* studies and drug testing. Co-implantation of PDX-derived tumor cells with stromal cells, such as cancer-associated fibroblasts (CAFs), in recipient mice creates ideal conditions for studying tumor–stroma interactions. Optimizing tumor sample selection, multi-site engraftment, and orthotopic modeling can further enhance implantation success, positioning PDX models for expanded applications in cancer research. PDX-derived platforms, including organoids (PDO/PDXO), cell lines (PDC/PDXC), and tumor slice cultures (PDE), enable rapid drug testing, complementing PDX models for foundational studies and drug response evaluations in CRC research.

Before PDX models, CRC diagnoses typically occurred at advanced stages, necessitating a combination of surgery, radiotherapy, and empirical chemotherapy, which often showed limited efficacy due to tumor heterogeneity. PDX models allow for personalized therapy, with PDX-guided drugs showing approximately 90% concordance with clinical outcomes with a sensitivity for the PDX drug screens of 96%, and specificity of 70%, significantly improving treatment efficacy.^14^^,^^63^ Besides, PDX models also help forecast drug resistance and identify effective therapies before clinical symptoms emerge.^14^^,^^91^ In summary, PDX models are accurate and clinically relevant tumor models for CRC research and personalized treatment.

## CRediT authorship contribution statement

**Yalan Lu:** Writing – review & editing, Writing – original draft, Funding acquisition, Conceptualization. **Xiaokang Lei:** Writing – original draft. **Yanfeng Xu:** Writing – original draft. **Yanhong Li:** Writing – original draft. **Ruolin Wang:** Writing – original draft. **Siyuan Wang:** Writing – original draft. **Aiwen Wu:** Writing – review & editing, Conceptualization. **Chuan Qin:** Writing – review & editing, Funding acquisition, Conceptualization.

## Funding

This work was supported by the 10.13039/100017053National Natural Science Foundation of China (No. 82303782), 10.13039/501100004826Beijing Natural Science Foundation (No. 7252096), the 10.13039/501100019005Young Elite Scientists Sponsorship Program by CAST (No. 2022QNRC001), and the Non-Profit Central Research Institute Fund of the Chinese Academy of Medical Sciences (No. 2023-PT180-01).

## Conflict of interests

The authors declare no competing interests.
